# A decision exercise to engage cancer patients and families in Deliberation about Medicare Coverage for advanced Cancer Care

**DOI:** 10.1186/1472-6963-14-315

**Published:** 2014-07-19

**Authors:** Marion Danis, Amy P Abernethy, S Yousuf Zafar, Gregory P Samsa, Steven P Wolf, Lynn Howie, Donald H Taylor

**Affiliations:** 1 Department of Bioethics, National Institutes of Health, Bethesda, Maryland, USA; 2 Sanford School of Public Policy, Duke University, Box 90253, Durham, NC 27708, USA; 3 Center for Learning Health Care, Duke Clinical Research Institute, Durham, USA; 4 Department of Biostatisitcs and Bioinformatics, Duke University Medical Center, Durham, NC, USA

**Keywords:** Medicare, Hospice benefit, Health priorities, Insurance benefits, Public participation

## Abstract

**Background:**

Concerns about unsustainable costs in the US Medicare program loom as the number of retirees increase and experiences serious and costly illnesses like cancer. Engagement of stakeholders, particularly cancer patients and their families, in prioritizing insured services offers a valuable strategy for informing Medicare coverage policy. We designed and evaluated a decision exercise that allowed cancer patients and family members to choose Medicare benefits for advanced cancer patients.

**Methods:**

The decision tool, Choosing Health plans All Together (CHAT) was modified to select services for advanced cancer patients. Patients with a cancer history (N = 246) and their family members (N = 194) from North Carolina participated in 70 CHAT sessions. Variables including participants’ socio-demographic characteristics, health status, assessments of the exercise and results of group benefit selections were collected. Routine descriptive statistics summarized participant characteristics and Fisher’s exact test compared group differences. Qualitative analysis of group discussions were used to ascertain reasons for or against selecting benefits.

**Results:**

Patients and family members (N = 440) participated in 70 CHAT exercises. Many groups opted for such services as palliative care, nursing facilities, and services not currently covered by the Medicare program. In choosing among four levels of cancer treatment coverage, no groups chose basic coverage, 27 groups (39%) selected intermediate coverage, 39 groups (56%) selected high coverage, and 4 groups (6%) chose the most comprehensive cancer coverage. Reasons for or against benefit selection included fairness, necessity, need for prioritizing, personal experience, attention to family needs, holistic health outlook, preference for comfort, freedom of choice, and beliefs about the proper role of government. Participants found the exercise very easy (59%) or fairly easy (39%) to understand and very informative (66%) or fairly informative (31%). The majority agreed that the CHAT exercise led to fair decisions about priorities for coverage by which they could abide.

**Conclusions:**

It is possible to involve cancer patients and families in explicit discussions of their priorities for affordable advanced cancer care through the use of decision tools designed for this purpose. A key question is whether such a conversation is possible on a broader, national level.

## Background

Design of health care benefit programs, especially national coverage schemes such as the United State’s Medicare program or the United Kingdom’s National Health Service, must account for competing pressures and interests, and high profile reform discussions in both nations are ongoing [1-4]. Optimal health is an obvious goal, but the design of benefit programs must be balanced by the cost of care. Other important considerations include the need for benefit packages of public programs to accommodate both the needs and preferences of large and diverse groups of patients and their family members, and to allow health care providers to treat their patients as they see fit within the professional norms of a given nation. As populations age and health care costs increase, a key question for western democracies is what health care benefit options should be provided as an entitlement in public programs?

Finding the proper balance between access to an expansive and flexible benefit package and the affordability of providing such a package is not a theoretical exercise, but a practical problem that is ideally addressed by obtaining the insight of a variety of perspectives, including those of patients and family members. But how and why is it important?

Administered by the U.S. federal government since 1966, Medicare is the national social insurance program that guarantees access to health insurance for Americans aged 65 and older who have worked and paid into the system (or who are married to someone who has), and younger people with disabilities. The benefit coverage decisions of Medicare have come to define not only those services to which the elderly are eligible to receive, but have evolved to practically define the standard of what care is “reasonable and necessary” throughout the entire health care system. Any change in what Medicare covers will influence the entire health care system [5,6]. People with cancer, and especially those with metastatic disease, have complex health care needs often requiring progressively more expensive cancer treatments (with unclear benefits and vague stopping points) superimposed on accelerating disease and treatment-related symptom burden as well as other needs requiring palliative intervention. From an insurance design standpoint, sorting out what could be provided and what is optimal to be offered is likely to be more difficult for cancer than for other diseases, in part because of the rapid increase in treatments without clear information about when they are warranted [7]. Finally, it is much simpler to state that there is a long run financing gap between expected expenditures and tax flows under current policy than it is to devise a different policy. For such a politically difficult task, the views of those most directly involved (e.g., those with cancer and family members) are needed to increase the chance of bringing about any consequential change in this area. In order to best devise policy, better information about the priorities of patients and family members dealing with cancer can be elicited using participatory decision tool processes.

The most compelling American approach to measure public preferences has been the Choosing Health plans All Together (CHAT) exercise, which has been used with over 4,000 lay participants in nine US states over a decade, to elicit public input in health insurance benefit design [8]. However, CHAT has only been used with members of the public without regard to whether they have a particular diagnosis. To date, it has not been used to ascertain the priorities of patients and families who have experienced or are in the midst of experiencing an illness.

This paper explores whether the CHAT exercise can successfully be used with cancer patients and families to reveal their perspectives in a way that can inform policy questions about what health care benefit options Medicare beneficiaries and their family members would prefer when faced with a resource constraint. If use of the CHAT exercise is of value to this patient population and the Medicare program regarding alignment of priorities, it may prove to be similarly useful with other patient populations in the United States or other nations interested in undertaking patient-centered health plan design, which is intended to maximize value for individuals and families so that they receive more benefit and better results for their health care dollars.

## Methods

### Participants

Study participants were cancer patients and family members of cancer patients recruited from the Duke Comprehensive Cancer Center, cancer clinics at Duke Raleigh Hospital, a variety of health care facilities, and organizations that serve a large number of senior citizens in the Raleigh-Durham, North Carolina area. This included continuing care retirement communities, organizations that provide support to cancer survivors, and non-profit organizations that aid families who are receiving health care from Duke University Medical Center. Two participants were excluded because of insufficient attendance at a CHAT session. One individual enrolled but chose not to complete participation.

### Use and modification of the CHAT exercise

**Figure 1:**
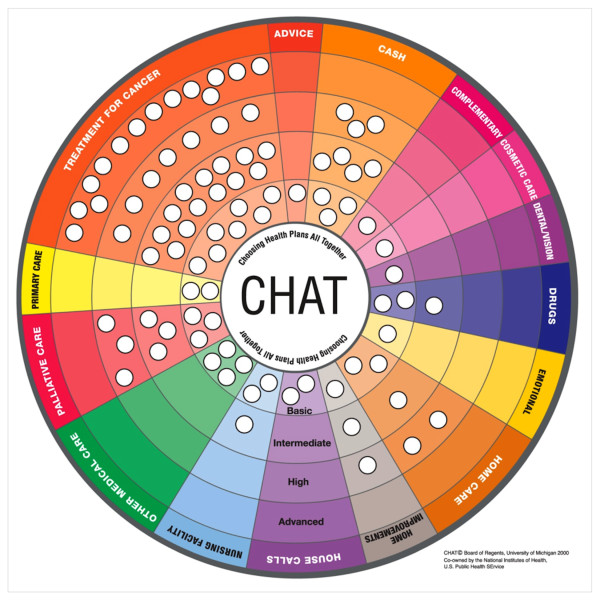
Advanced cancer CHAT board.

We used the Choosing Health plans All Together (CHAT) exercise to create a version for cancer patients and their families. CHAT is an interactive decision tool designed to facilitate deliberation by groups comprised of members of the public in order to select health plan benefits.The exercise uses a circular board on which various health plan benefits are represented as pie-shaped coverage options for selection by participants; the circular presentation avoids a more hierarchical presentation that might influence selection of options. Participants can choose benefits at as many as four levels of care and related expense depicted in concentric rings around the board (Figure 1). These levels generally offer increasing degrees of choice, convenience, and expanded services, and diminishing degrees of out-of-pocket costs.

Participants were each given 50 markers with which to select health plan benefits. These markers represented an amount of money comparable to the average annual expenditure by Medicare enrollees in 2007 as identified in Medicare claims files. The dollar value was inflated to 2010 dollars, the initial year of CHAT data collection. Each benefit category could be selected at any level by placing the specified number of markers on the board; the number of markers proportionally reflected the benefit’s actuarial cost. It would take 87 markers to choose the highest level of benefit coverage for all options, so participants faced a severe resource constraint in making their selections. Participants were not told the CHAT game was based on money, but were simply told they could not choose everything.

CHAT session participants sat at a table and used laminated paper exercise boards with stickers that allowed them to specify their insurance benefit choices. A trained facilitator guided participants through the exercise, answered questions, and therefore ensured participants had credible information about the choices before they stated their preferences.

Participants made choices about their benefit preferences in four rounds. During round 1, participants made benefit selections individually. In Round 2 they worked in small groups to select benefits by consensus. In Round 3, all participants decided upon a group benefit package. Finally, in round 4 they again made benefit selections individually.

At the outset of the exercise, the facilitator’s script explained to study participants that the purpose of the CHAT exercise was to help design a better Medicare health plan and to help people make informed health plan choices with a combination of affordable benefits that are compatible with the needs of patients with advanced cancer. The facilitator explained the need to prioritize health plan coverage options in the face of limited resources and advised participants to refer to the printed booklet of insurance benefit options (see Additional file 1 for booklet content). Unlike the usual CHAT exercise, participants were not instructed to make benefit packages for themselves but rather for advanced cancer patients in general. This instruction was specifically chosen because the study team knew neither the stage of participant’s cancer nor what these patients had been told about their disease by their oncologists, and were instructed to avoid pressuring participants to discuss their personal experiences of living with cancer with strangers in the group.

After Rounds 1 and 2, the facilitator answered questions and provided some examples of health events depicting experiences of advanced cancer patients to provide needed context for these discussions, and to facilitate informed and prudent choices. At the beginning of Round 3, the facilitator gave the following instructions about the deliberative process:

Now, just as the game says, we’ll “Choose a Health plan All Together” as one big group. To do this, we’ll use this Big Game Board *(Point to Board at the front of the room).*

You will each take a turn saying what you recommend and negotiate to get a group decision. First we’ll ask a player to recommend a category for basic coverage. If no one objects to what is suggested, I’ll place red stickers on the Big Exercise Board. If you don’t agree, let me know and we will discuss that category. After we’ve considered all categories for the Basic level of coverage, we can go back and look at Intermediate and High levels of coverage in the categories that are important to us. When we get to the last five stickers or so, the decision may get tougher. We’ll try to reach agreement. If there are services you want to give up to pick some other service that is fine.

We did not give study participants any specific instructions about how to take into account supplemental insurance coverage or their personal incomes. Rather we were interested in how they might spontaneously take these factors into account. We now mention this is the Methods section.

The original CHAT exercise was additionally modified as follows.

### Selection of benefit options

Since the aim of the exercise was to prioritize Medicare benefits that would offer affordability and address the goals of care for advanced cancer patients, a range of options were chosen that were compatible with the full dimension of services from which advanced cancer patients might possibly benefit. The major benefit categories (as shown in the online additional material) include domains that range from cancer treatments with management of treatment complications, to preservation of function, psychosocial support, logistical support, and financial support. The benefit categories offered as choices to participants were selected based upon published data about the health care needs of people with advanced life-limiting illness [9,10]. In addition, some of the benefits choices are not currently covered by Medicare: unrestricted cash that may not typically be viewed as a benefit in such a health care program, concurrent palliative care where expansive palliative services could be accessed without un-electing curative treatments as now required in the current Medicare hospice benefit, and in-home custodial long term care services to address disability.

### Estimation of actuarial cost of benefit options

Actuarial costs of the benefit options were estimated using the cost of Medicare financed care in the last six months of life for a claims-based cancer death cohort of Medicare decedents age 65 and older (5% claims sample, 2007), using the amount paid by Medicare. We identified decedents who had at least one hospitalization in their last six months of life with a primary cancer diagnosis (signified by one ICD-9-CM code in the following ranges: 140.xx-165.xx, 170.xx, 171.xx, 172.xx, 174.xx, 175.xx, 176.xx, 179.xx-195.xx, 200.xx-208.xx, 236.6×).

Detailed lists of typical drugs were developed for common types of cancer, and overall costs divided to represent basic, medium, high, and advanced benefit levels based upon average costs of care from Medicare claims. Prices used to finalize cost estimates of the different benefit levels came from Medicare reimbursement rates, from the literature, and from local market data for items such as family members paid time. We compared the Medicare reimbursement rates to the charges of Duke University Medical Center for other insurers for comparison purposes to ensure that they were generally aligned. Our cost estimates needed to be general because they were used to develop a CHAT tool for a Medicare sample facing diverse types of cancer. The goal was to provide a realistic cost vs. benefit tradeoff for patients and family members.

For those options that we included but do not exist in the current Medicare benefits, (discretionary cash, concurrent palliative care and expanded LTC), we used internal Duke University cost estimates as well as local market estimates to estimate the relative “cost” of these benefits.

Choosing the maximum level of all benefit categories would require 87 stickers with an estimated cost of $60,900; participants only had 50 stickers with which to choose. While the benefit options were broadly based on Medicare and other costs, monetary value of benefit options in the exercise was not explicitly communicated to CHAT recipients. Participants were simply told they only had 50 stickers and could not choose everything.

### Testing of the exercise

To determine participant assessment of the cancer CHAT exercise, we used a post exercise questionnaire (four-point Likert scales) that queried if participants thought the exercise was enjoyable, understandable, easy, and informative. Participants were also asked about their level of agreement with a number of attitudinal statements. Discussions during Round 3, in which full groups deliberated about and chose a benefit package, were audiotaped and transcribed for a subset of 20% the CHAT groups to ascertain the quality of deliberative process among this unique group of cancer patients and family members.

### Human subjects protection

The Duke University Health System Institutional Review Board (IRB) approved the study. The Office of Human Subjects Protection at the Clinical Center of the National Institutes of Health exempted the study from IRB review. Participants gave informed consent and, at the conclusion of the exercise, were given a check for $75 as compensation for their participation.

### Data analysis

Choices of categories and tiers and responses to pre- and post-surveys were summarized using descriptive statistics. Fisher’s exact test was used to test for significant differences between patient and family member demographics and choices that were collected during pre- CHAT surveys, while information on participant views of the experience were collected during post-CHAT surveys. Transcriptions were reviewed to ascertain the reasons that participants gave for their benefit selections and verbatim quotes illustrating keys themes were selected for inclusion in the online additional material.

## Results

### Participant characteristics

A total of 440 participants (246 patients and 194 family members) participated in 70 CHAT sessions. Target group size for CHAT sessions was 8-10 participants, but smaller groups were also held (smallest CHAT n = 3). Cancer patients were an average of 73 years of age, 49% were female, and 26% were African-American. Approximately half of participating patients had completed college (46%), 36% had annual household incomes under $40,000, and 50.4% had spent $2,000 or more out of pocket on medical care in the prior year. Patients had a wide range of cancer types. Family members were an average of 64 years of age. They were predominantly spouses (51%), but they also included adult children (19%), siblings (4%), and other adults (20%). There was a statistically significant difference between patients and family members for age, gender, marital status, educational attainment, health status and out-of-pocket expenses (Table 1).

**Table 1:** Socio-demographic characteristics and health status of patients (N = 246) and family members (N = 194)

**Characteristics**	**Patient (N = 246)**	**Caregiver (N = 194)**	**Combined (N = 440)**
**Mean age ± SD**^**b**^	73 ± 7.1	64 ± 10.7	69 ± 10.0

**Gender**^**b**^			
Female	120 (48.8)	138 (71.5)	258 (58.8)
Male	126 (51.2%)	55 (28.4%)	181 (41.1%)
Not Answered	0 (0.0%)	1 (0.5%)	1 (0.2%)

**Race**			
Native American	6 (2.5)	2 (1.0)	8 (1.8)
African American	64 (26.3)	67 (34.9)	131 (30.1)
Caucasian	169 (69.5)	120 (62.5)	289 (66.4)
Other	4 (1.6)	3 (1.6)	7 (1.6)
Not Answered	3 (1.2%)	2 (1.0%)	5 (1.1%)

**Ethnicity**			
Hispanic or Latino	2 (0.8)	1 (0.5)	3 (0.7)
Not Hispanic or Latino	219 (91.6)	167 (89.8)	386 (90.8)
Not Answered	25 (10.2%)	26 (13.4%)	51 (11.6%)

**Marital status**^**d**^			
Married/live with a partner	171 (69.5)	151 (78.2)	322 (73.3)
Single or never married	6 (2.4)	9 (4.7)	15 (3.4)
Widowed/Divorced/Separated	69 (28.0)	32 (16.6)	101 (23.0)
Not Answered	0 (0.0%)	2 (1.0%)	2 (0.5%)

**Relation to patient**			
Spouse		99 (51.6)	
Sibling		8 (4.2)	
Child		36 (18.8)	
Other		39 (20.3)	
Not Answered		12 (6.2%)	

**Educational attainment**^**d**^			
Less than high school	20 (8.2)	9 (4.7)	29 (6.6)
High school graduate or GED	43 (17.6)	45 (23.3)	88 (20.1)
Some college	66 (27.0)	69 (35.8)	135 (30.9)
College graduate or more	114 (46.7)	70 (36.3)	184 (42.1)
Not Answered	3 (1.2%)	1 (0.5%)	4 (0.9%)

**Household income**			
<20 K	39 (15.9)	27 (14.0)	66 (15.1)
20-39.9 K	49 (20.0)	32 (16.6)	81 (18.5)
40-59.9 K	48 (19.6)	46 (23.8)	94 (21.5)
60-79.9 K	49 (20.0)	34 (17.6)	83 (18.9)
80 + K	57 (23.3)	50 (25.9)	107 (24.4)
Not answered	4 (1.6%)	5 (2.6%)	9 (2.0%)

**Health status**^**a,b**^			
Poor	8 (3.3)	0	8 (1.8)
Fair	50 (20.3)	16 (8.3)	66 (15.0)
Good	93 (37.8)	75 (38.9)	168 (38.3)
Very good	70 (28.5)	73 (37.8)	143 (32.6)
Excellent	24 (9.8)	29 (15.0)	53 (12.1)
Not answered	1 (0.4)	1 (0.5%)	2 (0.5%)
			
**Cancer type (self reported)**			**Deceased N (%)**
Breast	43 (17.5)		2 (4.6)
Lung	38 (15.4)		10 (26.3)
Prostate	37 (15.0)		4 (10.8)
Other	26 (10.6)		6 (23.1)
Colon	17 (6.9)		
Lymphoma	14 (5.7)		
Bladder	11 (4.5)		
Pancreatic	11 (4.5)		
Head/Neck	7 (2.8)		
Ovarian	7 (2.8)		
Leukemia	6 (2.4)		
Multiple myeloma	6 (2.4)		
Endometrial	3 (1.2)		
Kidney	3 (1.2)		
Skin	3 (1.2)		
Brain	3 (1.2)		

**Out-of pocket payments during the past 12 months**			
$0	11 (4.5)	6 (3.1)	17 (3.9)
<$500	20 (8.1)	37 (19.1)	57 (13.0)
$500 - < $2000	55 (22.4)	34 (17.5)	89 (20.2)
$2000 or more	124 (50.4)	95 (49.0)	219 (49.8)
Not Answered	36 (14.6)	22 (11.3)	58 (13.2)

^a^Health Status was a patient’s response to the question: “In general, would you say your health is:”

^b^P < 0.001.

^c^P < 0.01.

^d^P < 0.05.

### CHAT groups’ coverage priorities

Over 60% of the CHAT groups chose coverage for cancer treatment at the two intermediate levels among the four levels that were offered. Many groups chose to include all other benefits that are not covered by the Medicare program including emotional support for patients and family members (53%), cash assistance (46%), advice (29%), house calls by physicians (27%), and home improvements (70%) (Table 2).

**Table 2:** Overall frequency (%) of responses to the post-CHAT questionnaire

	**Combined (N = 439%)**^**a**^
**Q1: Doing the CHAT exercise was:**	
1. Very enjoyable	318 (72.4)
2. Fairly enjoyable	100 (22.8)
3. Fairly un-enjoyable	5 (1.1)
4. Very un-enjoyable	9 (2.1)
N/A	7 (1.6)
**Q2: Doing the CHAT exercise was:**	
1. Very easy to understand	259 (59.0)
2. Fairly easy to understand	172 (39.2)
3. Fairly hard to understand	1 (0.2)
4. Very hard to understand	0
N/A	7 (1.6)
**Q3: Was doing the CHAT exercise:**	
1. Very informative	291 (66.3)
2. Fairly informative	135 (30.8)
3. Fairly uninformative	3 (0.7)
4. Very uninformative	2 (0.5)
N/A	8 (1.8)

^a^No statistical significance was found between family members vs. patient between these questions allowing us to pool the data.

### Qualitative analysis of group deliberations

The reasons that study participants gave to justify their benefit selections included arguments for the following: fairness, necessity, need for prioritizing, personal experience, attention to family needs, holistic health outlook, preference for comfort, freedom of choice, and beliefs about the proper role of government. A summary table showing verbatim quotes in support of or against each benefit option and which type of argument was used is shown in Additional file 2.

Because, there is such controversy regarding the extreme cost of some cancer medications, we reviewed those comments in which participants mentioned their reasoning regarding selection of the level of cancer treatment they endorsed. There was not substantial disagreement regarding selection of the level of cancer treatment and thus few statements justifying the levels selected; only 3 of 14 analyzed group deliberations involved comments about the level of cancer care selected (Additional file 3).

In contrast, **s**tudy participants did engage in discussing the financial ramifications of their benefit choices in general. In seven of the 14 analyzed sessions, participants made explicit statements regarding financial ramifications of benefit selections (range in the number of comments per session was 0-22 per session, see Additional file 4).

Because study participants were asked to consider what they thought advanced cancer patients’ coverage ought to be rather than to consider what coverage they would wish to have themselves, we examined how participants’ own experience contributed to their reasoning they gave during the deliberative process. Among the recorded sessions, 6 or 14 sessions included at least one comment explicitly referring to personal experience as a justification for the benefit selection that the participant was recommending. Sessions varied widely in the extent of such comments (ranging from 0 to 25 comments, see Additional file 5).

### Attitudes of study participants regarding the exercise

Reactions of patients and families regarding the exercise were similar and are therefore reported together (Table 3). Participants found the exercise very enjoyable (73%) or fairly enjoyable (23%); 2% found the exercise very unenjoyable. Participants found the exercise very easy to understand (59%) or fairly easy to understand (39%), and none found it very hard to understand. They also found the exercise very informative (66%) or fairly informative (31%; Table 3).

**Table 3:** **Overall frequency (%) of Likert responses to the post-CHAT questionnaire**^**a**^

**Survey item**	**1 Strongly disagree**	**2**	**3 Agree**	**4**	**5 Strongly agree**	**N/A**
Q4 When I think about CHAT I feel angry	312 (71.1)	34 (7.7)	43 (9.8)	16 (3.6)	29 (6.6)	5 (1.1)
Q5 When I think about CHAT I feel frustrated	276 (62.9)	61 (13.9)	49 (11.2)	17 (3.9)	25 (5.7)	11 (2.5)
Q6 The way the group reached its decision was fair	19 (4.3)	8 (1.8)	100 (22.8)	62 (14.1)	243 (55.4)	7 (1.6)
Q7 My views were considered and taken into account	15 (3.4)	4 (0.9)	91 (20.7)	68 (15.5)	255 (58.1)	6 (1.4)
Q8 During the CHAT I was treated with respect	16 (3.6)	2 (0.5)	55 (12.5)	47 (10.7)	310 (70.6)	9 (2.1)
Q9 What I wanted was considered by the group in arriving at a decision	16 (3.6)	8 (1.8)	84 (19.1)	77 (17.5)	249 (56.7)	5 (1.1)
Q10 Discussion during the CHAT was open and honest	17 (3.9)	2 (0.5)	72 (16.4)	46 (10.5)	300 (68.3)	2 (0.5)
Q11 Disagreements were resolved fairly	8 (1.8)	6 (1.4)	103 (23.5)	57 (13.0)	245 (55.8)	20 (4.6)
Q12 Information given to us was believable	11 (2.5)	9 (2.1)	105 (23.9)	75 (17.1)	223 (50.8)	16 (3.6)
Q13 We had enough information to make decisions	6 (1.4)	22 (5.0)	103 (23.5)	90 (20.5)	204 (46.5)	14 (3.2)
Q14 Choices given to us were realistic	10 (2.3)	17 (3.9)	107 (24.4)	89 (20.3)	195 (44.4)	21 (4.8)
Q15 My own choice of a plan is very different from what the group chose	135 (30.8)	138 (31.4)	60 (13.7)	52 (11.8)	32 (7.3)	22 (5.0)
Q16 All the choices I wanted were available to me	13 (3.0)	32 (7.3)	151 (34.4)	81 (18.5)	131 (29.8)	31 (7.1)
Q17 I was satisfied with the group’s decision	4 (0.9)	9 (2.1)	124 (28.2)	95 (21.6)	177 (40.3)	30 (6.8)
Q18 I would be willing to abide by the group’s choice of Medicare coverage	6 (1.4)	22 (5.0)	141 (32.1)	87 (19.8)	150 (34.2)	33 (7.5)
Q19 Asking cancer patients/families about what kind of Medicare benefits they recommend is a good idea	6 (1.4)	6 (1.4)	77 (17.5)	69 (15.7)	250 (56.9)	31 (7.1)
Q20 Having cancer patients and families talk about their views in a group was a good experience	8 (1.8)	6 (1.4)	81 (18.5)	64 (14.6)	252 (57.4)	28 (6.4)
Q21 I would rather not talk about the things we discussed today	270 (61.5)	74 (16.9)	26 (5.9)	14 (3.2)	25 (5.7)	30 (6.8)
Q22 I would like to have insurance for patients with cancer be spent on medical care only	186 (42.4)	102 (23.2)	59 (13.4)	25 (5.7)	38 (8.7)	29 (6.6)
Q23 I would like to have insurance for cancer patients cover broader needs than medical care alone	21 (4.8)	34 (7.7)	108 (24.6)	76 (17.3)	170 (38.7)	30 (6.8)
Q24 The presentation was effectively organized	4 (0.9)	4 (0.9)	75 (17.1)	65 (14.8)	259 (59.0)	32 (7.3)
Q25 The facilitator communicated effectively	6 (1.4)	1 (0.2)	64 (14.6)	63 (14.4)	275 (62.6)	30 (6.8)

^a^Not all participants answered every question.

The majority of participants agreed with statements indicating that the CHAT exercise led to fair decisions about priorities for coverage, took individual points of views into account, involved open and honest discussion, involved realistic choices, and led to group decisions by which they could abide (Table 3). They also agreed that asking cancer patients and their families about what kind of Medicare benefits they recommended was a good idea. There was distribution across the full range of opinions, however, and small but vocal minorities of patients can receive outsized attention. For example, even though nearly 8 in 10 respondents answered 1 (strongly disagree) or 2 (disagree) to the question “When I think about CHAT I feel angry” 6.6% strong agree with this statement. In discussions of health care policy changes such as the recent health reform discussion in the U.S., vocal minorities receive a great deal of attention making any consequential change difficult. One key question that is telling in this regard is the distribution of responses to the question “I would be willing to abide by the group’s choice of Medicare coverage” which only had around one-third of participants saying strongly agree, and a similar proportion providing the answer of 3-right in the middle.

The consensus benefit choices made at round 3 (Table 4) show what options rose to the top when a benefit constraint was imposed. Several choices are worth noting. First, only 4 groups (of 70) choose the maximum level of cancer treatments even though Medicare commonly finances such a level of “do everything” care if patients so desire. On the other hand, no groups choose the most minimal level of cancer treatment implying that these were not whimsical choices. Rather, they understood the reality of cancer for patients but were still willing to engage in tradeoffs. Second, nearly half of all groups selected some level of unrestricted cash, a benefit not provided by Medicare. Similarly, 32 groups selected home care (custodial long term care) at levels beyond what Medicare currently covers and 33 selected one of two levels of concurrent palliative care.

**Table 4:** Frequency (%) of consensus groups (Consensus round 3) choosing selected coverages (N = 70 groups)

**Coverage**	**0**	**1**	**2**	**3**	**4**
Treatment for cancer			27 (38.6)	39 (55.7)	4 (5.7)
Primary care	1 (1.4)	69 (98.6)			
Palliative care	4 (5.7)	33 (47.1)	30 (42.9)	3 (4.3)	
Other medical care	10 (14.3)	60 (85.7)			
Nursing facility	5 (7.1)	27 (38.6)	38 (54.3)		
House calls	51 (72.9)	19 (27.1)			
Home improvement	21 (30.0)	34 (48.6)	12 (17.1)	3 (4.3)	
Home care	5 (7.1)	33 (47.1)	22 (31.4)	10 (14.3)	
Emotional	33 (47.1)	37 (52.9)			
Drugs		11 (15.7)	59 (84.3)		
Dental vision	13 (18.6)	57 (81.4)			
Cosmetic care	19 (27.1)	51 (72.9)			
Complementary	50 (71.4)	20 (28.6)			
Cash	38 (54.3)	19 (27.1)	10 (14.3)	3 (4.3)	
Advice	50 (71.4)	20 (28.6)			

Coverage: 0 = no coverage, 4 = highest level of coverage.

## Discussion

Worries about the sustainability of public benefit programs due to rising costs, and the countervailing pressure to provide expansive benefits to seriously ill patients, is a seminal, and shared public policy conundrum across the industrialized world. The issue in the case of cancer is particularly explosive due to the rapidly increasing therapy options and the understandable inclination of patients to want more treatment options. How do public benefit programs say no to expensive, low value therapies?

We describe the modification and testing of a decision tool for use by patients and families intended to address this policy problem. The paper reports the consensus benefit choices of elderly cancer patients covered by the Medicare program in the U.S. (an entitlement program that covers all elderly persons) and their family members, arrived at after making individual judgments and smaller group discussions. These consensus benefit choices are made by persons who have actually been touched by cancer-either as a patient or as a family member. Those directly affected by this serious illness provide a key perspective to consider when determining what benefit options are provided to beneficiaries suffering from the common diagnosis of cancer. A notable finding is how infrequently these groups (N = 4) chose the advanced level of cancer treatment (that would require allocation of 42 of 50 stickers to cancer treatment), even though this level of care is commonly provided in the U.S. Medicare program. Instead, the consensus choices tended to provide for more balanced benefits, including palliative care and long term care services that address disability and the burdens of illness. For example, 32 of 70 groups allocated some resources to unrestricted cash-a benefit not provided in the current Medicare benefit package. Such selections came at a direct reduction of currently covered benefits, and demonstrate that persons who have experienced cancer were willing to engage in difficult tradeoffs that sometimes would result in allocating scarce resources away from medical treatments only in favor of a more balanced allocation. Similar shifts toward non covered benefits were identified in concurrent palliative care and home based long term care.

A limitation of the study is that it is a hypothetical exercise-the choices made in the CHAT exercise did not influence actual Medicare benefits, but the aim of the CHAT exercise was to impose a resource constraint that does not currently exist. The results of the study suggest that individuals who have experienced or been touched by significant illness such as cancer and thus might be quite affected by insurance benefit decisions may be prepared to explicitly confront the hard decisions inherent in high cost-high stakes medicine that have been thought to be so unapproachable if they are guided by carefully designed decision aids like the CHAT exercise.

Given the plethora of therapies and uncertainty of benefits in many cases of advanced disease, a key issue is the need to reset the expectations of each patient, family members, and providers regarding how to determine what treatments are provided in public benefit programs for cancer. The results reported here demonstrate that it is possible to facilitate such conversations with those individuals who are likely to face the brunt of the consequences of the insurance coverage decisions that will ensue. The study does point out, however, differences between conclusions that people arrive at from group deliberations in theoretical situations and the care that is provided by the Medicare program as a matter of course to patients with advanced disease such as cancer. For example, no groups chose the most advanced level of cancer care offered in this exercise, but the Medicare program routinely finances the ‘do everything’ version of cancer therapy for beneficiaries. This contrast highlights a profound separation between a Medicare enrollee’s understanding of the issues in the abstract when asked to consider the policy issues related to resource constraints and inclinations when actually receiving care under coverage of the Medicare program. Of course, patients do not receive the type of information and opportunity to discuss these difficult decisions in a facilitated manner in the normal functioning of the Medicare program. A key issue for all industrialized nations is to determine how to bring about meaningful discussion of the tradeoffs between types of benefits to inform the need to make these programs fiscally sustainable.

The need to thoughtfully consider how to set limits on the expensive care of advanced cancer patients is not merely a concern for the Medicare Program. The American Society for Clinical Oncology has recognized that the costs of cancer care are burdensome for patients and their families and has recommended that oncologists discuss the costs of cancer treatments with their patients. Fojo and Grady [11] as well as Smith and Hillner [12] have argued that the spiraling cost of cancer care, and particularly cancer therapeutics that achieve only marginal benefits, pose the need for guidance from oncologists about how to address these costs. Yet there has been little opportunity for cancer patients and their families to provide input into priority setting for benefit coverage for cancer care of advanced cancer patients. Our results suggest that when a structured process that allows for informed deliberation is conducted, cancer patients and their families are able and willing to explicitly discuss the burdensome costs of cancer care, the opportunity costs they pose, and their preferred benefit choices under the difficult circumstances they face. Much work remains to be accomplished in identifying strategies to translate this understanding into practice. To be effective, any new strategies will need to handle patient and family expectations in ways that allow for a financially sustainable coverage while at the same time maintaining patients’ confidence that they are receiving appropriate care.

This study has several limitations. First, like most focus group studies that involve recruitment of individuals who must be available for sessions that may not convenient for all those eligible, this study includes participants who are not a representative sample of elderly cancer patients. Nonetheless, the study population does include individuals with varied ethnicity, income and education. Second, as stated earlier, the exercise posed a hypothetical set of choices. We cannot infer with certainty that participants would engage in such a process as willingly or make the same choices if they were posed in circumstances where the consequences were more concrete.

## Conclusion

Our results suggest that this CHAT exercise can be used with cancer patients and their families to determine the nature and scope of Medicare benefits that would be meaningful to this clinical population. Exercises like this can be helpful to inform policy questions about the alignment of Medicare benefits specific to patients with advanced cancer. Given the favorable responses from our sample, extension of the CHAT exercise may prove to be useful with other patient populations to help guide policy makers interested in patient-centered health plan design.

## Competing interests

Dr. Donald H. Taylor received research funding from US Agency for Healthcare Research and Quality and the Robert Wood Johnson Foundation, the Duke Endowment, National Institute for Nursing Research, National Cancer Institute, National Institute on Aging, and the Center for Medicare and Medicaid Innovation (CMMI). He has also received support from the Robert Wood Johnson Foundation for blogging at the Incidental Economist, from the Diabetes Care Group, Inc., and has been a paid speaker for Blue Cross/Blue Shield and the Carolinas Center for Quality of Care.

Dr. Abernethy has research funding from the National Institute of Nursing Research, National Cancer Institute, Agency for Healthcare Research and Quality, DARA, Glaxo Smith Kline, Celgene, Helsinn, Dendreon, Kanglaite, Bristol Myers Squibb and Pfizer; these funds are all distributed to Duke University Medical Center to support research including salary support for Dr. Abernethy. Pending industry funded projects include: Galena and Insys. Since 2012, she has had consulting agreements with or received honoraria from (>$5,000 annually) Bristol Myers Squibb and ACORN Research. Dr. Abernethy has corporate leadership responsibility in athenahealth (health information technology [IT] company; Director), Advoset (an education company; Owner), and Orange Leaf Associates LLC (an IT development company; Owner). She is pending employment with Flatiron Health.

Dr. Zafar is supported by an American Cancer Society Mentored Research Scholar Grant and the Duke Cancer Institute Cancer Control Pilot Award.

Drs. Samsa, Howie, and Mr. Wolf have no competing interests to disclose.

Dr. Danis receives a small portion (<1%) of her salary from royalties derived from the CHAT exercise under a copyrighted held by the University of Michigan and shared with the National Institutes of Health.

## Authors’ contributions

DHT was the study supervisor who provided administrative, technical or material support, acquired the data, had full access to all of the data in the study, and takes responsibility for the integrity of the data and the accuracy of the data analysis. DHT and APA obtained the funding and designed the study. GPS and SPW performed the statistical analyses. DHT, GPS, and SPW analyzed and interpreted the data. DHT, MD, SYZ, and APA drafted the manuscript and provided critical revisions for important intellectual content. LH reviewed patient records and charts and assigned cancer type and stage in conjunction with SYZ. All authors have read and approved the final manuscript.

## Pre-publication history

The pre-publication history for this paper can be accessed here:

http://www.biomedcentral.com/1472-6963/14/315/prepub

## Supplementary Material

Additional file 1 **The CHAT booklet that contains benefit descriptions and definitions as well as the number of stickers required (i.e., the ‘cost’ to the patient) to receive basic, intermediate, high, and advanced level of care for each benefit.** Referenced in the text as Additional file 1. Click here for file

Additional file 2 **The reasoning (for and against) that study participants gave to justify their benefit selections.** Referenced in the text as Additional file 2. Click here for file

Additional file 3 **Verbatim quotes from participants regarding Level of Cancer Treatment coverage.** Comments on financial ramifications were presented in three of the 14 analyzed sessions. Referenced in the text as Additional file 3. Click here for file

Additional file 4 **Verbatim comments from participants about the financial ramifications of benefit choices.** Comments on financial ramifications were presented in seven of the 14 analyzed sessions. Referenced in the text as Additional file 4. Click here for file

Additional file 5 **Verbatim comments from participants about how their own personal experience contributed to their reasoning in the deliberative process.** Comments on personal experiences were presented in six of the 14 recorded sessions. Referenced in the text as Additional file 5. Click here for file
