# Supplementary material for: A decision exercise to engage cancer patients and families in Deliberation about Medicare Coverage for advanced Cancer Care
Source: BMC Health Serv Res. 2014 Jul 19;14:315. doi: 10.1186/1472-6963-14-315 (PMC4112612; doi:10.1186/1472-6963-14-315)
Supplement: Additional file 1 — The CHAT booklet that contains benefit descriptions and definitions as well as the number of stickers required (i.e., the ‘cost’ to the patient) to receive basic, intermediate, high, and advanced level of care for each benefit. Referenced in the text as Additional file 1. [file 1472-6963-14-315-S1.docx]

**Additional File 1**

**ADVICE:** Insures that a person gets help with writing a living will or other legal papers so the family and doctor will know what to do if the patient is not able to make decisions. A person may also get help with planning to distribute personal property. This may include preparing a will and setting up trusts to transfer one’s assets. This can reduce taxes after death.

###### **Basic:** (1 Sticker) A person receives the services described above.

**CASH:** This provides cash to patients to spend as they need. For example, a person can pay rent or mortgage, medical care, medicines, food, travel, or homemaker services.

###### **Basic:** (3 Stickers) A person receives $360 each month.

**Intermediate:** (7 Stickers) A person receives $840 each month.

###### **High:** (10 Stickers) A person receives $1200 each month.

**COMPLEMENTARY:**  This provides alternative services. This includes acupuncture for pain, chiropractic, massage treatments and relaxation treatment. It also includes visits to a spa or a retreat.

**Basic:** 1 sticker A person receives the above treatments and services.

**COSMETIC CARE:** This insures services to help cancer patients recover how they look and look their best.

###### Basic: (1 Sticker) A person gets needed surgery, wigs, prostheses, scar treatment and scar repair, and other treatments.

**DENTAL/VISION:** This helps patients living with cancer to pay to prevent problems with their teeth or eyes. It also pays for tests and treatments for dental and visual problems. This includes contact lenses and eyeglasses.

#### Basic: (1 Sticker)

- A person receives regular cleanings and X-rays.
- A person has cavities filled and bad teeth extracted.
- A person receives complete dental care including crowns.
- A person receives an eye exam every two years.
- A person receives lenses and frames if needed.

**DRUGS:** This provides help to people living with cancer to pay for non cancer-related prescribed medicines. Cancer medicines would be provided under the “Cancer Care” category.

###### **Basic:** (2 Stickers) A person has part of their outpatient prescribed drugs paid for as in Medicare Part D.

**Intermediate:** (3 Stickers) A person has all outpatient prescribed drugs paid for. This covers an insurance gap and is called “doughnut hole” coverage.

**EMOTIONAL SUPPORTS:** This provides support and counseling, support groups, and pastoral care. This also covers support for family members during treatment and after a cancer patient dies. Psychosocial services can be provided to patients and family members, either alone or together. The goal of such services is to help patients and family members cope with the effect of cancer on the patient and family.

###### **Basic:** (1 Sticker)

- A team includes nurses and social workers. It also has grief counselors and pastoral counselors for psychological needs of patients and family members.
- The team also gives information about and referrals to religious and spiritual family members for patients and families.

##### **HOME CARE:** This covers medical services at home by licensed health care workers. This can provide nursing, to get drugs and other treatments. These can include intravenous and aerosol treatments, tube feeding, and other medical treatments. It can also include health aide services to help with bathing, toileting, feeding, and other personal care.

###### **Basic:** (2 Stickers) All in-home care is paid. Each month a patient receives:

-4 visits from a nurse and

-Daily care from a nurses' aide for 1 hour

**Intermediate:**  (3 Stickers) All in-home care is paid. Each month a patient receives:

-8 visits from a nurse and

-Daily care from a nurses' aide for 2.5 hours

**High:** (5 Stickers) All in-home care is paid. Each month a patient receives:

-15 visits from a nurse and

-Daily care from a nurses' aide for 2.5 hours

**HOME IMPROVEMENTS/EQUIPMENT:**  This provides items such as wheelchairs, walkers, and raised toilet seats to assist persons with limited ability to move on their own. It can also provide home remodeling including ramps, stability bars and lifts to assist with moving in the patient’s home and to help getting in and out of bed.

###### **Basic:** (1 Sticker) A person receives needed equipment for use at home, including walkers, canes, wheelchairs, and commodes.

**Intermediate:** (2 Stickers) Includes the above equipment plus bathroom and/or living area remodeling to assist with activities of daily living.

###### **High:** (3 Stickers) Includes the above plus outside improvements to a person’s residence.

**HOUSE CALLS:** This insures a medical provider such as a physician, nurse, or physician assistant can come to the patient’s home to diagnose any medical problem or complaint.

**Basic:** (2 stickers) A person receives the above care if needed.

**NURSING FACILITY:** Pays for a skilled nursing home or rehabilitation center. This provides short term skilled nursing care and related services for patients. This can provide services to improve the function of injured, disabled, or sick persons. Such facilities provide all care needed by patients.

###### **Basic:** (2 Stickers) A person receives help with daily needs and skilled care prescribed by doctors provided by nurses and certified nurse assistants.

**Intermediate:** (3 Stickers) Includes the above plus physical, occupational, and other therapy.

**OTHER MEDICAL CARE:** This provides for hospital care to address major health problems that may be unrelated to cancer. This may include heart surgery following a heart attack, hip or knee replacement to address mobility disability, or surgery to address a chronic back ailment. This covers services such as x-rays, laboratory tests, major surgery, and follow-up outpatient

###### **Basic:** (4 Stickers) A person receives the above treatments and care if he or she experiences any of these events.

**PALLIATIVE CARE:** This provides relief of pain, stress and any other troubling symptoms of serious illness.

**Basic:** (3 stickers) Consists of the standard Medicare hospice benefit that exists today. This includes a team approach to pain and symptom management, psychosocial support for patient and family members including bereavement counseling for family members, if a patient chooses to stop receiving therapy that is designed to forestall or cure cancer.

Care is provided by a multidisciplinary team that includes nurses, social workers, dieticians, pastoral care, nutritionists, and physicians.

Care will typically be provided in the patient's home, though inpatient hospice is available.

**Intermediate:** (6 stickers) Consists of the above treatment, plus

*occasional respite care to allow family family members to have a break; and consultation from a doctor who is a pain specialist, even while the patient continues to receive anti-cancer therapy. This physician works with cancer doctors to treat pain and other symptoms along with their cancer care.

**High:** (9 stickers) Consists of above treatment, plus

*the same multidisciplinary team that provides hospice care would provide care throughout the entire course of a patient’s cancer treatment.

You could get all of these services even while receiving treatment to stall or cure cancer

*an advice line available 24 hours a day, 7 days a week, and a home visit if needed, at any point during treatment. If a home visit is needed, a nurse will arrive at a patient's home within 1 hour of being called, 24 hours a day/7 days a week; and

* all services are available regardless of what type of treatment is being received for cancer.

**PRIMARY CARE:** This provides first contact care for persons to diagnose any sign, symptom, or health concern. This is the care provided by a patient’s typical health care provider. This would take care of illnesses that might be unrelated to their cancer.

###### **Basic:** (2 Stickers) A person receives office visits, outpatient services (non-cancer) and preventive services.

**Treatment for Cancer:** Cancer care is given to control a patient’s cancer. It treats cancer symptoms. It manages treatable medical problems that happen during cancer treatment.

**Basic:** 7 stickers.  Basic cancer care is defined as treatment that is very cost-efficient, may somewhat improve quality of life, may have many bad side effects, and is not provided to patients who are in bed more than half of their waking hours.  Advanced cancer patients receiving Basic cancer care have an average lifespan gain of 2 months.  Out of 100 patients treated with Basic cancer care, 1 will have their lifespan increased by 6 months.

**Intermediate**: 21 stickers. Intermediate cancer care is defined as treatment that is cost-efficient, may improve quality of life, may have a number of bad side effects, and is not provided to patients who are in bed more than half of their waking hours.  Advanced cancer patients receiving Intermediate cancer care have an average lifespan gain of 3 months.  Out of 100 patients treated with Intermediate cancer care, 1 will have their lifespan increased by 18 months.

**High:** 28 stickers. High cancer care is defined as treatment that is not very cost-efficient, is likely to improve quality of life, has few bad side effects, and can be provided to patients whether or not they are in bed more than half of their waking hours.  Advanced cancer patients receiving High cancer care have an average lifespan gain of 4 months.  Out of 100 patients treated with High cancer care, 1 will have their lifespan increased by 3 years.

**Advanced:** 42 stickers. Advanced cancer care is defined as treatment that is not cost-efficient, may somewhat improve quality of life, may have a number of bad side effects, and can be provided to patients whether or not they are in bed more than half of their waking hours.  Cancer patients receiving advanced cancer care have an average lifespan gain of 6 months.  Out of 100 patients treated with advanced cancer care, 1 will have their lifespan increased by 5 years.
