# Supplementary material for: A decision exercise to engage cancer patients and families in Deliberation about Medicare Coverage for advanced Cancer Care
Source: BMC Health Serv Res. 2014 Jul 19;14:315. doi: 10.1186/1472-6963-14-315 (PMC4112612; doi:10.1186/1472-6963-14-315)
Supplement: Additional file 2 — The reasoning (for and against) that study participants gave to justify their benefit selections. Referenced in the text as Additional file 2. [file 1472-6963-14-315-S2.docx]

Additional File 2

| **Category** | **Reasons for** | **Reasons Against** | **General Discussion** | **Quotes** | **Values** |
| --- | --- | --- | --- | --- | --- |
| **Treatment for Cancer** | (Session A): Hope that the chances are actually better than the ones given | (Session A): Chances given are not that great | (Session A): Statistics might be better than the ones given; people have seen patients live for much longer | (Session A): "I would hate to deny somebody who needs that level of care" (High) | Fairness |
|  | (Session B): It's a necessity; cost-efficient and may improve quality of life (as it's defined); can go through treatment pretty quickly; could live longer than expected | (Session B): You can spend a lot on treatment but then not have great quality of life because you didn't spend on other things; only gets you marginal improvements in length of life | (Session B): Some people like to look at the periphery of coverage before getting into treatment | (Session B): "We know we're gonna have to have that- a lot of it;" | necessity/valuable |
|  | (Session K): Personal experience with treatments not being paid for because they are rare; advanced treatement covers research like clinical trials | (Session D): not worth it for just 3 months- question of what you're trying to achieve- living at all costs no matter the side effects/money? Personally, wouldn't want to live a long time in a feeble condition; at that age, even healthy people can't do much; high only gives you 4 months (just 1 more than intermediate); expensive** | (Session I): does this include research? | "It is effective in terms of extending life and you can pretty quickly get through that first level of treatment" (Intermediate) |  |
|  | (Session D): High treatment might represent breakthroughs for the general population (experimentation, etc.) | (Session F): Little indication that certain treatments are effective in the long run |  | (Session K): "We know that's what the majority want" (Intermediate) | necessity |
|  | (Session G): Most important thing | (Session G): Uses up a lot of stickers- would miss out on important things |  | (Session D): "Do you want to live at all costs...no matter how many hundreds of thousands of dollars it takes to get you to live 3 more months?...For me, it's clearly not worth it" (Intermediate) | Prioritizing (*against* things- not worth it) |
|  | (Session H): has personally seen/experienced treatment | (Session M): Requires a lot of stickers/money |  | (Session G): "I think the care for cancer is the most important thing.” |  |
|  |  |  |  | "That's what's helped my husband" (Intermediate) |  |
|  | (Session M): thinks "it's important;" "you want your money there" |  |  | (Session M): "You want as much treatment as you can" (Advanced) | necessity/valuable |
|  | (Session I): if we don't have it, we'll die; it's necessary; has heard more and more stories of complications/unforeseen treatments coming up |  |  | (Session J): "More treatment, and better treatment, is necessary;" | personal experience |
|  | (Session N): it's necessary for people who need it; it's just important; it's expensive; some people need it; personal experience of needing more treatment than expected |  |  | "I was on an experimental chemo program...90 day supply of the medication was $38,000. And my insurance company picked it up" (Intermediate) | necessity |
|  |  |  |  | (Session N): "It's very necessary for the people who need it;" | necessity |
|  |  |  |  | "It's just important, you know, the cost...the patient may just need it;" |  |
|  |  |  |  | "I don't have cancer cells but they're wanting to put me on this or that or one thing or another.” | personal experience |
|  |  |  |  | "If they said, well...you're gonna wind up in a rest home...yeah I'd probably say no to it" (Intermediate/high- recording cut off) |  |
| **Primary care** | (Session A): It's a necessity |  |  | (Session A): "The primary physician is the very basic necessity for good healthcare" | Necessity |
|  | (Session B): Seen by the primary physician more than anyone else; physician can even become a close friend |  |  | (Session B): "We’re looking at the periphery before we get into the treatment, and you can get in there and spend all of your money on treatment and not have very much quality of life;" | holistic health outlook; |
|  | (Session C): thinks "it's important" |  |  | "Two stickers, that's not that much" | cost efficient |
|  | (Session E): Need primary care even if you don't have cancer (preventive services- mammograms, etc.); primary care is usually the first to catch problems |  |  | (Session E): "Preventive screenings like mammograms and other screenings...I think that's critical" | necessity/valuable |
|  | (Session F): Treats all other things (hypertension, post-cancer treatment); only two stickers; usually the place that diagnoses |  |  | (Session G): "That's where a lot of people first find out they've got a problem" | necessity/valuable |
|  | (Session G): Gets everything outside of cancer; where a lot of people are first diagnosed |  |  | (Session I): "You do get sick and you have other things going on with you" | other health issues |
|  | (Session I): you'll still get sick; rely on general practitioner |  |  | (Session N): "If you're getting your checkups...you'll have a head start" | necessity/valuable |
|  | (Session N): if you're getting checkups, you can get a head start |  |  |  |  |
| **Palliative care** | (Session A): Respite for family is a good feature- the experience can be rough/you need a break/someone will come relieve you |  |  | (Session A): "Respite for the family members and that's really rough when you're there and you need a break" | consideration for family/caregivers |
|  | (Session B): Ability to give family members a break through respite care (this is very important to her); long-term hospice at home helps the caregiver |  |  | (Session B): "I've seen situations in the hospice facility but I've also seen hospice at home where, and it's a long term thing that would really help the caregiver" | consideration for family/caregivers; personal experience |
|  | (Session C): thinks "it's important;" emotional issue |  |  | (Session C): "It's all emotional stuff for me" | holistic health outlook |
|  | (Session D): Important when you’re living a long time- most people need 6 months hospice; treatment gets counterproductive- at some point you just need comfort; intermediate level helps caregivers; paying for morphine administration could be expensive on your own |  |  | (Session D): "Most of us probably will have about 6 months of end of life care that's needed." | holistic health outlook |
|  | (Session E): Covers a lot of emotional things; want to have overall quality of life; living an additional amount of time in chronic pain/fatigue isn't necessarily good |  |  | "Treatment gets counterproductive after a while, but you do need a certain amount of comfort 'til you die" | necessity/valuable |
|  | (Session G): Not necessarily only end of life care; respite care for family is important |  |  | (Session E): "That covers lots of stuff related to emotional things;" | holistic health outlook |
|  | (Session I): get a lot out of it |  |  | "That's a big thing there" | valuable |
|  | (Session I): important for the patient to have access to such a team; cancer patients will ultimately end up in that position and need help; gives respite to caregiver when patients are bedridden |  |  | (Session I): "You get a lot out of that one" | cost efficient |
|  |  |  |  | (Session I): "With cancer patients, ultimately you're going to walk that path, and you're gonna need help;" | necessity/fairness |
|  |  |  |  | "I think sometimes for patients who are in bed a lot of the time...the opportunity for the caregiver to have a little rest is important" | consideration for family/caregivers |
| **Other medical care** | (Session A): Cancer is not the only disease; a lot of people on a fixed income can't pay for everything; could get into trouble; can have two things go wrong |  |  | (Session A): "A lot of people on fixed income can't pay for any other additional…" | fairness |
|  | (Session B): Quality of life; everyone losing hips/knees |  |  | (Session C): "That one's important" | necessity/valuable |
|  | (Session L): Can have diseases outside of cancer; personal experience with heart problems |  |  | (Session I): "With my treatment, it's probably strong and I'll need other medicine to help keep my blood pressure down or whatever other side effects I might have" | necessity; personal experience; other health issues |
|  | (Session C): amazing amount of services |  |  |  |  |
|  | (Session D): cancer isn't typically the only health issue; might need knee replacements, people fall, etc. |  |  |  |  |
|  | (Session G): Need coverage for things outside of cancer |  |  |  |  |
|  | (Session I): will need other medicine to help deal with side effects of cancer treatment |  |  |  |  |
| **Nursing facility** | (Session A): Personal experience with Nursing Facilities; people think physical therapy is the most important thing (included at intermediate level) |  |  | (Session A): "Having personal experience with the nursing facility--not for me but for family--I think I'd like to put two green circles on the basic level of nursing;" | personal experience |
|  | (Session B): Can help you with occupational things (getting out of chairs, going to the kitchen, etc.); without attention, people might fall and have to start the healing cycle all over again; people with hip/knee replacements need physical therapy |  |  | "Physical therapy, that's the most important thing there" | holistic health outlook |
|  | (Session K): intermediate level therapy can help quality of life/mobility |  |  | (Session B): "If you're sculpting out a Medicare policy here, you wouldn't want the person that had the hip replacement or the knee replacement left out of physical therapy and a nursing home" | fairness |
|  | (Session C): "it's important;" therapies can be effective--story of a friend |  |  | (Session K): "Physical, occupational, and other therapy which will never help me if I'm 70 years old...but it will help somebody young." | holistic health outlook |
|  | (Session D): Expensive if it's not covered (personal example); most people need some kind of therapy (personally uses all three) |  |  | "But it could help somebody older have better quality of life...mobility of hands and limbs..." | fairness |
|  | (Session E): Facilities (particularly after surgery) are good because you're under constant care with regular rehab; objective caregivers; could help you get independent/back home sooner |  |  | (Session C): "You've got to have a transition for somebody who's really serious in the hospital;" "There are a lot of people who need that transition level" | holistic health outlook |
|  | (Session F): Sometimes caregivers are actually in a bad enough condition that they can't help |  |  | (Session D): "I believe I do all three [therapies]" |  |
|  | (Session H): need physical/occupational/other therapy |  |  | (Session E): "I think for two stickers, that's pretty reasonable" | fairness |
|  | (Session I): provides skilled nursing care; more specialized (need it through Medicare, not other sources); more long-term than other things; not everyone has family members to care for them; might be in a condition not conducive to home care; might require nursing care |  |  | (Session F): "We've situations where...people are themselves so bad off that they couldn't care for..." |  |
|  | (Session I): it's important; if you don't have a family member, need somewhere to go/someone to take care of you; a lot of people today need that care; can speak to you on an easier level than doctors |  |  | (Session H): "Most people...are gonna need physical, occupational, and other therapy, so you're gonna deprive them of that?" | personal experience |
|  |  |  |  | (Session I): "Nursing gets expensive" | cost efficient |
|  |  |  |  | (Session I): "If you don't have a family member, you need to have somewhere to go and someone to help take care of you, especially if you're not able to do it yourself | fairness, necessary/valuable |
| **House calls** | (Session B): In the future we will be cared for by PA's and nursing experts; might make sense for some patients who are homebound and can't get transportation; could get a good relationship going with someone when they are coming to your house on a regular basis | (Session B): Don't know of doctors that make house calls; people usually have a way of getting to the doctor; opening a new can of worms; have to think in terms of the whole plan (not cost-effective); not cost-effective | (Session B): Could possibly be covered under Home Care or Palliative Care; HIPAA rules and regulations make it difficult to bring people to your house as much | (Session B): "It might really make sense for some patients who just can't, you know they're homebound, they can't get transportation out" | Fairness |
|  | (Session C): some people get sick enough that they can't leave | (Session D): Aren't productive |  | (Session C): "Some people are going to be sick enough…" | Fairness |
|  | (Session D): Good for people who are too sick to leave home; only "if needed" so no unnecessary calls; might need to stay home if you're on oxygen; family can't always take you to the hospital | (Session H): not actually for treatment, just diagnosis |  | (Session D) "If you have oxygen or you're housebound for some reason…" | Fairness |
|  | (Session H): some people can't get to doctor/live in rural areas where transportation is a challenge | (Session I): not a priority; hard to get physicians to come to your house; more cost-effective to go to the doctor |  | (Session H): "Some people can't get to the doctor... people who live out in rural areas... transportation is a challenge" | Fairness |
|  | (Session N): personal experience with needing her doctor at home |  |  | (Session I): "I don't think we need house calls, because a family member can take you there or you have a medical transport" | prioritizing against other options |
|  |  |  |  | (Session N): "I have a doctor and for procedures and things I need in my home, I have to call her and get her to sign for it and I really need it" | personal experience, necessity |
| **Home improvement, equipment** | (Session B): In favor of people staying at home to get better; tools that you need at home are in the coverage; people might need things installed at home; has witnessed that such equipment allows people to have quality of life and recover or spend the rest of their time in their home | (Session B): Don't know of doctors that make house calls; people usually have a way of getting to the doctor; opening a new can of worms; have to think in terms of the whole plan (not cost-effective); not cost-effective | (Session G): Like cash- is Medicare the proper venue? | (Session B): "I'm all for people staying at home and getting better" | comfort; holistic health outlook |
|  | (Session K): Some patients need equipment | (Session D): Aren't productive |  | (Session C): "Raised toilet seats- those do make a difference if someone has a problem" | fairness |
|  | (Session D): Equipment is importance; might need it for a knee replacement; second level- Medicare might not cover everything; makes life easier | (Session H): not actually for treatment, just diagnosis |  | (Session F): "If you can't live in your own home and be able to take a bath because you don't have the right equipment…" | comfort; holistic health outlook |
|  | (Session F): Necessary; bad if you can't take a bath/live in your own home; only one sticker | (Session I): not a priority; hard to get physicians to come to your house; more cost-effective to go to the doctor |  | (Session I): "There are groups that will provide home improvement" | prioritizing against other options |
|  | (Session G): Wheelchair access is important; only one sticker |  |  | (Session I): "Makes it more convenient for the person;" | necessity |
|  | (Session H): if you want to stay at home, may eventually need things like shower on first floor |  |  | "A lot of times you need those items and if you have to go buy them they are not cheap" | cost-effective |
|  | (Session J): need the items but they are not cheap; makes things more convenient |  |  |  |  |
| **Home care** | (Session B): Cost effectiveness: High coverage could keep a patient out of a nursing home, which is far more expensive; this could serve the need for house calls | (Session K): Could get home care through palliative care (hospice) | (Session B): Could it be abused by someone? | (Session B): "Many, many times, that home care is keeping the patient out of a nursing home, which is far more expensive, so from a cost-effective point of view…(trailed off)" | cost-effective; comfort; fairness |
|  | (Session C): thinks "it's important" |  |  | (Session K): "The reason I didn't go with that, I though maybe we could get some of that out of home health...in hospice?" | other options/prioritizing |
|  | (Session E): From a caregiver's perspective, it's nicer to have them at home; more comfortable for the patient and easier on the family; difficult to transport to the hospital; hospital trips can be long even if you aren't treated for a while; could help you get to the point where you can leave the house for visits; an objective individual at home is priceless because they can tell the patient what you can't; if you need someone bathed/a wound cleaned, you need a trained person |  |  | "There are some charitable organizations that do provide things like that" |  |
|  | (Session F): Personal experience of taking care of mother-in-law: helpful to have a break; can be expensive to hire someone to relieve the caregiver |  |  | (Session E): "From a caregiver's perspective, it's so nice the more that you can have them in the home...it's easier on the family...it was always hard to get in the car" | valuable; consideration for family/caregiver; personal experience |
|  | (Session H): personal desire to stay at home; have to have somebody at home; could give some respite |  |  | (Session F): "Having worked with her mother when she was in her last years...it was just so nice to be able to get away occasionally" | personal experience; consideration for family/caregiver |
|  | (Session I): already had her surgery, wants to stay at home; second level only one more sticker |  |  | (Session H): "I wanna stay home;" "You'd actually be giving some respite" | comfort; consideration for family/caregiver |
|  | (Session I): so I can get some help if I need it; being at home feels better than being in a strange hospital; easier for family to stop by; nice to have someone around; helps you stay at home longer |  |  | (Session I): "I've already had my surgery. I want care at home" | personal experience |
|  |  |  |  | (Session I): "They'll feel like they're getting better if their in their own house and their own surroundings than being in the hospital or being into a strange area;" "I like to have somebody around me at times" | holistic health outlook |
| **Emotional support** | (Session F): important to some people to be in support groups for example | (Session I): someone else can support you |  | (Session C): "I think the emotional support...is actually far more important...than the actual treatment that the medical people put it" | holistic health outlook |
|  | (Session G): Not everyone has a strong family that can help support you |  |  | (Session F): "I think that's important to some people…I didn't see any need for one…but I think a lot of people do need that" | fairness |
|  | (Session H): from breast cancer perspective, covers a lot; emotional/quality of life issue |  |  | (Session G): "Not everybody has that kind of help" (referring to a supportive family) | fairness |
|  | (Session I): personal experience with getting cancer twice; counseling from an independent person can be very helpful; can help your family; only one sticker |  |  | (Session I): "When you get hit with it the second time, I fell apart...and, like I said, by coming to someone who was independent and used to counseling was able to help me get stabilized;" | personal experience |
|  | (Session N): cancer affects everyone in the family; sometimes you just need someone to talk to |  |  | "That' s only one sticker" | cost effective |
|  |  |  |  | (Session N): "Cancer affects everyone in the family;" | family |
|  |  |  |  | “Sometimes that person who has cancer needs someone to talk to" | holistic health outlook |
| **Drugs** | (Session B): There are a lot of people who are kept back because of drugs they need; expensive; Part D of Medicare only covers a certain amount; experimental drugs are expensive; with cancer as a background, the opportunity to spend a lot on drugs is certainly real |  |  | (Session B): "I feel there are a lot of people that are kept back because of the drugs that they need;" | fairness; personal experience; |
|  | (Session L): Can be very expensive; insurance doesn't always cover everything |  |  | "My brother has a melanoma, he went to a dermatologist just on Monday, went to get the prescription filled and it was an $800 tube of medicine." | necessity/valuable; |
|  | (Session C): expensive; personal experience of expensive drugs |  |  | “Think of people who don't have coverage" | cost effective |
|  | (Session D): Issue of donut hole- lack of coverage |  |  | (Session E): "Drugs are expensive" | necessity/valuable; |
|  | (Session E): Drugs are expensive |  |  | (Session F): "Drugs by themselves can cripple a family" | necessity/valuable; |
|  | (Session F): expensive; can cripple a family financially |  |  | (Session G): "I wanted to make sure we didn't miss that" | necessity/valuable; |
|  | (Session G): Don't want to miss out on this coverage- important |  |  | (Session I): "Drugs are so darn expensive when you buy them on your own, and you got to have them" | necessity/valuable; |
|  | (Session H): expensive |  |  | (Session N): "It goes along with treatment, they're gonna prescribe some time of, you know, drug, or something that they're gonna need for the treatment" | necessity/valuable; |
|  | (Session M): Medicare doesn't cover the donut hole |  |  |  |  |
|  | (Session I): personally out of drugs; second level only one more sticker |  |  |  |  |
|  | (Session I): expensive to pay on your own; you'll have side effects from cancer treatment which make you need drugs; medication costs a lot; covers the donut hole |  |  |  |  |
|  | (Session N): goes hand in hand with treatment, often prescribed drugs with treatment |  |  |  |  |
| **Dental, vision** | (Session A): Experience of losing teeth with Chemo; a lot of people don't have this (and participants think they should) | (Session C): Unrelated to the cancer treatment |  | (Session A): "I lost my teeth with chemo;" | personal experience; |
|  | (Session B): Medicine can blur people's eyes even if your eyes are good; people need equipment that they can use to read | (Session D): Not currently covered by Medicare- and with Medicare's current issues with affordability, shouldn't try to make them cover more; this coverage is fairly restrictive anyway |  | "I think a lot of people don't have it and I think they should" | fairness |
|  | (Session K): Medications can sometimes have an impact on your teeth/eyes |  |  | (Session B): "So many people, their medicine blurs their eyes…people do need equipment that they can read with" |  |
|  | (Session C): thinks "it's important" |  |  | (Session D): "Eye care is pretty important;" "You gotta see" | necessity |
|  | (Session D): Eye care is pretty important; need to see |  |  | (Session E): "Most older people end up needing cataract surgery...if you don't have the extra cash, you're in trouble." | fairness |
|  | (Session E): Most older people end up needing cataract surgery; need to get your teeth cleaned/to get screenings; 1 sticker goes a long way |  |  | "It's cheap with the one sticker" | cost effective |
|  | (Session G): Good for seniors; "if it's there, let's take it" |  |  | (Session G): "Great need for seniors" | necessity/valuable; |
|  | (Session H): covers cavities, crowns, eye exams, glasses; teeth may be damaged during chemo; it is a quality of life issue |  |  | (Session H): "That's quality of life issue" | holistic health outlook/quality of life |
|  | (Session M): important for everyone to have dental/vision; especially when you get older and need glasses |  |  | (Session M): "I think it's important that everybody has dental and vision, especially when you get to my age and you got to have some glasses" | fairness, necessity |
|  | (Session I): eye glasses are expensive; depending on how incapacitated you become, your dental care is going to suffer |  |  | (Session I): "Depending on how incapacitated you become...your dental care is going to suffer for it" | fairness, necessity |
| **Cosmetic care** | (Session A): Only one sticker; important | (Session F): Can get this from other places- personal experience with Susan G. Komen | (Session C): Shouldn't just be labeled as cosmetic | (Session A): "It's only one sticker" | cost-efficient; holistic health outlook |
|  | (Session B): Women may need wigs; men can understand what it means for a woman to get her hair fixed | (Session I): Should maybe expect people to have a little cash to pay for these things- not integral to your life, just how you feel, more aesthetic; should spend money on more important things (in this case, nursing) | (Session I): Can maybe get cosmetics somewhere else? | (Session B): "I know what it means to a woman to get her hair fixed" |  |
|  | (Session C): Important to women with breast cancer; an emotional issue; makes you feel more normal; intertwined with emotional; treatment is about dealing with the whole person |  |  | (Session C): "To me, cancer is about treating the person…and you actually deal with the problem that [cancer] has created with the person" | holistic health outlook |
|  | (Session D): quality of life improvement |  |  | (Session D): "If you're well enough to get to that point of cosmetics, it will improve your quality of life" | fairness; holistic health outlook |
|  | (Session F): For people who need wigs after chemo |  |  | (Session F): "There are other ways to pay for it" | prioritizing (*against* it)/other options |
|  | (Session I): not just wigs; friend had to pay out of pocket |  |  | (Session H): "That's a quality of life issue" | holistic health outlook/quality of life |
|  | (Session I): important after mastectomy; wigs and other things; psychological issue; makes a difference when you can take care of yourself; more positive perspective on life; personal/anecdotal experience of having a positive perspective |  |  | (Session I): "Shouldn't I have a little cash in my pocketbook to handle an unforeseen thing like a wig?" |  |
|  |  |  |  | "It isn't integral to your...existence" | prioritizing (*against* it)/other options |
|  |  |  |  | (Session I): "Mastectomy is very very, very important;" | necessity |
|  |  |  |  | "They teach you how to look healthy again;" | holistic health/quality of life; |
|  |  |  |  | "It has a lot to do with your psychological approach to it, your mindset" | holistic health/quality of life; |
| **Complementary** | (Session A): Things like getting a massage/being pampered can make you feel like a million dollars; relieves stress; doesn't work for everyone, but neither does medicine! | (Session A): Acupuncture doesn't work for everyone; people don't like the wording of "spa;" excessively spending money |  | (Session A): "To be pampered can make you feel like a million dollars" | holistic health outlook |
|  | (Session B): Has used complementary services (acupuncture and chiropractic services) for 15 years/has kept her and her husband healthy | (Session C): Massages are fun but not worth the money; "spa" treatment doesn't seem medical |  | (Session B): "I use complementary services--acupuncture and chiropractic--and so does my husband, for 25 years, and has basically kept us healthy" | personal experience |
|  | (Session K): Recent news that massages are good for cancer patients and other illnesses; can help the medical aspect; know of people who have been helped by chiropractors, acupuncture | (Session E): Can be covered through cash |  | (Session K): "They just said on the news how good massages are for cancer patients;" | holistic health outlook |
|  | (Session C): Suffering needs "feel-good" treatment/pat on the back; this is intertwined with emotional support |  |  | "I think there are things that help the medical part of it;" | holistic health outlook |
|  | (Session D): Personally uses acupuncture/chiropractic services; sometimes you have back issues and need work on it |  |  | "I've heard a lot of people say acupuncture helps them with pain" | personal/anecdotal experience |
|  | (Session G): Personal experience of surgery that needed pain relief/blood flow through rehab, but rehab doesn't pay for massage therapy/acupuncture |  |  | (Session C): "Personal suffering needs that little pat on the back, you know, the feel-good treatment;" | holistic health outlook |
|  |  |  |  | "It's not a lot of money in the overall scheme of things" | cost effective |
|  |  |  |  | (Session D): "It includes chiropractic and acupuncture, both of which I use, so...for me I would spend one;" | personal experience |
|  |  |  |  | "It's just one sticker" | cost effective |
|  |  |  |  | (Session G): "I have to go to a massage therapist...but it comes out of pocket" |  |
| **Cash** | (Session B): Someone with practically no cash will need some; giving patients a choice of what to use their money for; could be more cost-effective | (Session B): Medicare isn't really responsible for helping you pay rent, food, travel; thinks cash will be abused; no control of how it's spent | (Session G): Seem to agree that people might need cash, but this is not the place for it | (Session B): "Put ourselves in the situation where this is someone who doesn't have--have practically no cash or no income;" | fairness |
|  | (Session E): Gives you the option of deciding which types of treatment to get/not to get; can manage individual needs; can cover things (like drugs) that Medicare doesn't pay for | (Session C): Mistake to give people cash; can't control spending |  | "At least it's putting choice in people's hands, instead of being told how to spend their money;" | freedom of choice |
|  | (Session F): Medicine alone could need that money | (Session G): Should not be part of a health program; makes you think of welfare/food stamps which is not Medicare; high chances of fraud; shouldn't come into play here; would rather see it go somewhere else; no way to document spending |  | BUT "Is Medicare really responsible for helping us pay our rent and our mortgage?" |  |
|  | (Session G): Can lose jobs with diagnosis, cash helps; maybe not appropriate for Medicare, but thinks people need cash | (Session H): not needs-based: even people who don't need it will get it; no way to track where it goes (moderator) |  | (Session C): "It's a mistake to give people cash;" | control of coverage |
|  | (Session H): people need cash, esp. if they lose their job; if you're paying for it, you're entitled to it (counterargument to needs-based system); only for people with cancer diagnosis; people not on Medicare could really be hurt without cash |  |  | "Look what they do with food stamps!" |  |
|  | (Session I): if an individual is diagnosed with cancer and isn't able to work/provide for their family, they'll need help; don't know how long someone will be sick; helps the family |  |  | (Session E): "It's more individualized to what your needs are;" | freedom of choice |
|  |  |  |  | "I've been healthy up until and...if there was cash I could have used it for some dental and some vision..." | personal experience |
|  |  |  |  | (Session G): "I think their [people who have lost their jobs] ability to continue on is dependent on getting a little extra help;" | fairness |
|  |  |  |  | BUT "I think cash should not be part of a health insurance program;" |  |
|  |  |  |  | "Chances of fraud are so big" | control of government; control of coverage |
|  |  |  |  | (Session H): "I don't need it but if somebody loses their job because they had cancer, and they have nothing;" | fairness; necessity |
|  |  |  |  | BUT "Anybody could get it" |  |
|  |  |  |  | (Session I): "It depends upon if the individual is diagnosed with cancer and they're not able to work and they're the bread-winner of that household, they need somewhere to live, they need food;" | fairness |
|  |  |  |  | "You don't know exactly how long an individual's going to be sick" | fairness |
| **Advice** | (Session B): Importance of writing a living will; physicians can't quite give you legal advice | (Session B): Should Medicare be telling you what to do (write a living will); not the government's role; could possibly get advice from primary care | (Session B): Note- Medicare not telling you what to do but helping if you need help | (Session B): "The living will, which is so, so important;" | necessity/valuable |
|  | (Session F): Might not have faced this issue until now | (Session F): Should have already done this a while ago |  | BUT "I don't think it's the government's role, I think it's the individual's role" | control of government |
|  | (Session G): pretty important | (Session I): you can get this somewhere else (church), shouldn't have to rely on insurance; could be covered in palliative |  | (Session K): "You can get that advice from several other areas" |  |
|  |  |  |  | (Session F): "Sometimes...living wills and stuff like that, a lot of people don't face til they get to this position" | other options/prioritizing (*against)* |
|  |  |  |  | (Session I): "To me, that's free. If you're a church-going individual...I think that's something that we...shouldn't have to rely on our insurance to pay for;" | other options/prioritizing (*against)* |
|  |  |  |  | "Palliative care covers that" | other options/prioritizing (*against)* |
