# Supplementary material for: A decision exercise to engage cancer patients and families in Deliberation about Medicare Coverage for advanced Cancer Care
Source: BMC Health Serv Res. 2014 Jul 19;14:315. doi: 10.1186/1472-6963-14-315 (PMC4112612; doi:10.1186/1472-6963-14-315)
Supplement: Additional file 3 — Verbatim quotes from participants regarding Level of Cancer Treatment coverage. Comments on financial ramifications were presented in three of the 14 analyzed sessions. Referenced in the text as Additional file 3. [file 1472-6963-14-315-S3.docx]

Additional File 3

| **Session** | **Verbatim Quotes Regarding Level of Cancer Treatment Coverage** |
| --- | --- |
| Session D | “It could get you maybe 3 more months – is it worth it? My decision is made on the basis of my age. For me it’s clearly not worth it, both in terms of age and because I don’t want to live a long time in feeble conditions”  “Treatment gets counterproductive after a while, but you do need a certain amount of comfort until you die, and hospice gives you that”  “What confuses me about the way this is expressed is, if you look in intermediate, ‘*patients receiving intermediate care have an average life span gain of three months*,’ that kind of weird. You get 1 more month if you have high treatment, and you get 2 more months if you get advanced care…is it really worth it?”  Moderator: “So do we want to come back to that one? We still have enough money that we can come back to it, but we want to move on to something else, and then if somebody else decides later on we want to go there” (Agreement) |
| Session B | “I would like to address the cancer treatment. And of course looking at the intermediate, which would be 21 stickers, but, when you read the description and its says, ‘*intermediate cancer care is defined as treatment that is cost efficient, and improve the quality of life.’* You know, to me, as long as it’s cost efficient..”  Moderator: “Now let me stop you really quick, we don’t even have enough stickers for that one. So we only have – what do we have, 12 stickers – we have 12 left, and that would be 13 stickers, but – either we don’t put them there and we use them elsewhere or, you can move stickers from another spot to be able to fill treatment for cancer, but that’s based on what you guys want”  “Let’s look at advice again. Can you get advice from you primary, since we already have the primary doctor?”  “Well we could take two off house calls. We discussed that kind of long and hard anyway. I’m sorry, home care – I was reading that wrong.”  “I’d like to jump back in the picture now on with her on the treatment of cancer. Because it does say that it’s cost effective, and it is effective in terms of extending life, and you can pretty quickly get through that first level of treatment and be faced with the issue, ‘*do I go back?*’ We have a neighbor who just made that decision last week, to continue another 2 months of chemotherapy. I’m going to bring him back over here tomorrow for it, and I agree with him totally. In his case I would have gone after it too, so I think we want to endorse that second level”  “You extend your life 3 months…I personally don’t buy it, but – because the quality of lie at that stage isn’t that great anyway, usually. I think, let people make their own decisions without everybody being extended”  “Well it’s hard to know whether that three months is going to be quality or, it could be quality and then you bite the dust quickly or maybe part of it…So it’s an average number and you can’t, it’s hard to say”  Moderator: “You could live 3 more years”  (decide to remove from home care to get treatment) |
| Session A | Woman 1: “I want to go treatment for cancer second level”  Man 1: “I struggle with their explanation here. It makes no sense to me. It says it’s based on research, it says ‘*intermediate care is defined as treatment that is cost efficient, may improve quality of life, may have a number of bad side effects, and sis not provided to patients who are in bed more than half of their waking hours. Patients receiving intermediate cancer care have an average life span gain of 3 months. So that says half of them live longer than three months, half of them live less. Out of a hundred patients treated with intermediate cancer care, one will have their life span increased by 18 months.*’ So it says with the majority of people, is it even worth it? For 99 people, they’re not even going to live 18 months…Well is that really true?”  Other Woman: “I don’t think this is valid at all because after 18 months, you know, always in research, there is always new drugs coming up. And so, with this, we’re just saying, you know, this is the way it is.”  Moderator: “It’s just for the purpose of the research study because we can’t, we can’t include everything”  Man 2: “See then you go into high level, and it says you may or may not be restricted to bed 50% of your time, but you’ll have quality of life”  Moderator: “It’s up to you guys. If you think it’s worth the 18 months. If you’re one of the, 18 months – you might be more, you might be less – to spend the money”  Man 1: “I’m just torn. Well I’m for treatment; I just find t hard to believe that, if that’s the effect of regular treatment, they need more alternative treatment. If 99% of the people aren’t going to make it 18 months”  Other woman: “Well I would hate to deny somebody who needs that level of care, or personally even the next level of care. Because what I would be doing would be forcing them into palliative care or some other thing with my stickers”  Man 2: “See I question the data. Where is this 1/100…”  Man 1: “What I’m struggling with is when I read what they say, I just can’t believe what they’re saying is correct”  Man 2: “When you read the medical journals, we have a lot more success than this… so this statement right here doesn’t seem to sit well”  Man 1: “I don’ t think this is correct, and I think we should go to the intermediate and even higher…My wife has had it for 19 or 20 years now, so I mean I’m not a researcher, but I see all kinds of people. And if I thought it was only going to work for 18 months for 99% of the people, it’s like, you’ve got to be kidding me.“  Moderator; “I agree, and it’s good that we have this on recording. But because the research is based on this book, let’s do it based off of this. So if this was the truth, let’s do it based on this.  Other Woman: “There’s hopelessness built into that” (agreement)  Other Woman: “We’re voting for hope!” |
