# Supplementary material for: A decision exercise to engage cancer patients and families in Deliberation about Medicare Coverage for advanced Cancer Care
Source: BMC Health Serv Res. 2014 Jul 19;14:315. doi: 10.1186/1472-6963-14-315 (PMC4112612; doi:10.1186/1472-6963-14-315)
Supplement: Additional file 4 — Verbatim comments from participants about the financial ramifications of benefit choices. Comments on financial ramifications were presented in seven of the 14 analyzed sessions. Referenced in the text as Additional file 4. [file 1472-6963-14-315-S4.docx]

Additional File 4

| **Session** | **Verbatim Quotes Regarding Financial Issues** |
| --- | --- |
| Session C | “I don’t believe in giving people in cash, unless they have a savings account medical thing. But then if they got cash they shouldn’t be getting all the other services”  “It’s a mistake to give people cash” |
| Session D | “Well it’s not covered by Medicare now. Medicare is in trouble in terms of being affordable. And I think it’s a mistake to make Medicare cover more than it already covers…just makes matters worse” |
| Session E | “I would like to put two levels in cash…and possibly once we’re through put more into cash. And the thought process there, Marianne, I had in our conversation was these other things if you have the cash - some need one, don’t need this, may need more of that, may need more of whatever; so if you had the cash, you can manage that and you can get your wheelchair, you can go have extra care, or get cosmetic or go get emotional so you can better manage. Like a health service account, a health savings account…”  So it’s more individualized to what your needs are.”  “That’s a very good look at it”  “I’ve been healthy up until now and I figured if there was cash I could use it for some dental and some vision and some who knows what. And he has had a lot of health costs and even at that, like drugs- Medicare will just say, ‘*well we don’t cover that*.’ They won’t say why, they won’t give you any recourse, they just say, ‘*well we don’t cover that.*’ And I’m thinking we have this optimum drug coverage, and yet we fall in the donut hole. We have these problems, you know - but I f I had cash, I would just put it in, put it where the need exists” |
| Session I | Woman 1: “We’re talking a program, a government taking care of doing these things. I’m really just not sure they’re the ones that should be providing me a wig, I mean, really, shouldn’t I have a little cash in my pocketbook to handle an unforeseen thing like a wig…or be bald”  Woman 2:“Not if you’re on a fixed income”  Woman 1:“If you haven’t planned ahead, you get to skip the wig and other people don’t. That’s all I’m saying. It isn’t integral to your life being, to your existence. It’s integral to how you feel about yourself, I suppose”  Woman 1:“You know what sometimes you have to spill a little of your own money, you know just have to plan for a lousy end and put aside some money so you can buy a wig or whatever as opposed to giving up something else”  Woman 3:“It’s prosthesis, too”  Woman 4:“Are there other societies or organizations that would help with that expense?”  Woman 2: “We just had someone to go through that and she had that out of pocket…I know Medicaid won’t take care of it”  Woman 1:“There are organizations that fill in whenever there is a gap. So if the government all the sudden said, ‘*we’ll not do any treatment of cancer*,’ somebody will start filling in. You know, it’s just, where isn’t money being spent by the government, somebody will pop in for it. But where are the biggest expenses? Leave them with government.” |
| Session J | “I think I would go to cash. I would vote to put 3 on cash for the simple fact that it depends upon the individual diagnosed with cancer; and they’re not able to work and they are the bread winner of the household, they need somewhere to live, they need food, so that’s why I would go with cash“  “Drugs are so expensive, and if you need anything to…we all know once you start taking cancer treatment you get so many other side affects that causes you maybe to maybe have to have other drugs. And drugs are so darn expensive when you buy them on your own, and you’ve got to have them”  “I think we should go up to another level on drugs, only because of the costs of medication”  Woman 1:“I think I would move and put more on cash because you don’t know exactly how long an individual is going to be sick. It’s like after the first couple of months and your insurance runs out early, what do that bread winner do? What do the family do? I would like to go cash. And I have to move 2 stickers”  Woman 2:“You could take 1 off of homecare and 1 off of drugs”  Woman 1: “That’s what I would do”  “Drugs can make you go to the poor house very quickly”  “I work at the senior citizens center, and those folks are really very poor. And the older you get, it seems like the more problems you have, the more meds you need. They’re choosing to buy a pill that they can cut in half and not eat lunch. I mean they’re making major decisions, so drugs – drugs from where I sit – if drugs could just be solved for the world that would be a godsend for people”  “This is the United States of America, and that’s not right”  “I have discovered with the Blue Cross and Blue Shield plan, there are so many different umbrellas within the program. Back in the older times, the policies covered so, so much, but the new policies that’s coming out today, they say one thing but it don’t cover it because you have this, and this is why you don’t qualify for this. Because it don’t cover this piece of the puzzle”  “If I go to the doctor and say I have a headache, it’s well you’ve had a stroke and your insurance policy doesn’t cover a stroke …so it covers pieces of it. “  “One individual takes the x-ray, another individual reads the x-ray, and then another person tells you what it said.“  “One reason I want to be a part of this study is just to see what the outcome is…I would love to know what effect this study is to help insurance companies see what they’re charging us for. Especially for the public”  “I work for the state, and I have the damndest insurance plan in the world. And Each year it kept going up, up, up“  “Hers is 7 dollars, mine is 15. Same type of medicine, same brand name”  “We all know Medicare is going to be changing, they keep squeezing it, squeezing it, squeezing it…and I’m curious as to, they keep squeezing it and then you’re losing doctors that will support Medicare so then you can’t go to the doctor. You’ve got secondary insurance, but secondary insurance only pays after Medicare pays and that ball of wax is starting to unravel too”  “I hope this study actually won’t just be another study, with a dead end result”  Moderator: “The idea is that when this is published it will become public knowledge…your opinions matter here. And hopefully we’ll be able to use it to at least get a discussion going about what is wrong with the current system”  “I’m really concerned that the government has decided to keep cutting back on Medicare and to the doctors. The doctors are starting to opt out of taking patients with Medicare, so where is that really going to leave us all – with no doctors? It’s getting tough. I mean I’ve had friends who have had to change doctors because the doctors have dropped Medicare”  “They aren’t covering their expenses. The payment that they get from the government is so low compared to if you have private insurance going in, the doctors are losing money”  “What do you think about that healthcare they passed, that Obama passed? So many people don’t have insurance; I think it would help a whole lot of people in the world. But now they going to try to come back and undo it”  “It will help a lot of people who do not have insurance, it will…I don’t think, I don’t know, I don’t think they just want to undo the whole thing, I think they want to undo parts of it…it’s going to be a hard process for a little money”  “I think regardless of what happens you will see preexisting conditions honored. In other words you’re not going to be kicked out because you have a preexisting. I think everyone across the country, between all the policy makers agree that that was a good element of the package” |
| Session H | Man 1: “Cash. Somebody is going to need cash”  Woman 1: “Oh that’s right, I forgot all about cash”  Woman 1:“Yeah they’re going to need the cash instead of home improvement”  Man 2: I agree, there’s only 350 dollars a month on that basic, that’s not very much”  Woman 2: “Is it needs based? Somebody is going to be getting a nice big check every month from other things, are they going to be able to get that money too?”  Woman 1: “Well we got to think about everybody, we talking about the majority”  Man 2:“I don’t need it, but somebody who loses their job because they get cancer, and they have nothing”  Woman 2:“Yeah I’d like to see it though, to just to go to them and not to go to you, but anybody could get it, just like with social security, anybody gets it”  Man 2: “Fortunately I have a good pension, but I get social security. However my entire working career I’ve paid the maximum into social security, so that’s the trouble; if you’re paying into it, you should be entitled to it”  Woman 1: “But cash should be one of the main things”(Disagreement) |
| Session B | “I think the way we’re going, I kind of like because we’re looking at the periphery before we get into the treatment. You can get in there and spend all your money on treatment and not have very much quality of life because you didn’t take care of the other things, so I would kind of proceed along the line…I mean I know we’ll get there and spend some money on it. For example, primary care. All those things have to go on, and that’s not much money. 2 stickers on primary care. Our primary physician is one of our best friends. We see him more almost than anybody. I definitely would put two stickers on that” (Agreement).  “Well I think we out to start with the treatment, because we know we’re going to have to have that, a lot of it”  “I would fill that first level up as a starter...When I said as a starter, I didn’t mean we shouldn’t look beyond that”  “Home care you might need to go all the way to high because many, many times that home care is keeping the patient out of a nursing home, which is far more expensive. So from a cost effective point of view, if you went with the maximum, well you might not even have to choose between one and the other; you might be able to do both”  “I guess I was just looking at it from, it will keep them out of the nursing home, which is more expensive”  Man 1: “We haven’t talked about cash, and that’s a tough one because I assume that one is on Medicare, and f they’re on Medicare they’re probably on social security – you don’t know for sure. So we just try to put ourselves, as we talked about earlier as a group, put ourselves in a situation where this is someone who doesn’t’ have, have practically no cash, or no income. And if they do succeed in living another year or 2 - albeit maybe at home, in a bed or whatever- they’re going to need some cash so we put in – what did we put in, I think we put about 3 on that”  Woman 1: “I guess I have to take the negative side of this because this just, we’re talking Medicare; and is Medicare really responsible for helping us pay our rent and our mortgage, food, travel, you know whatever. And no. And I’m not sure whether this isn’t cash, when cash is going to get abused…There’s no control.”  Moderator: “It doesn’t say that they’re making you fill out a log or turn in receipts or anything”  Man 1: ”It opens the door. You’re right. But, if you’re expecting to succeed in keeping people in their homes as opposed to nursing homes, responsible for themselves…”  Woman 2: “Maybe choosing a helper that costs less than a service provider, you know how much it costs to bring a service person into…”  Man 1: “I think generally I’ve heard at least, that you can do that and stay in one’s home as opposed to paying 6 or 7 or 8 thousand dollars a month in a nursing home.”  Moderator: “It would literally be cash”  Other woman: “At least it’s putting choice in peoples’ hands, instead of being told how to spend their money”  Man 1: “Let’s just say we’re a worker. And we’re facing the fact that this person can’t – even if they own their home – lets’ just say they can’t. You can’t exist without cash. I mean you’ve got to be able to buy things to eat. You’ve got to have food, you’ve got to…If I’m a worker and I’m facing the fact that I’m going to move you out of your home and put you in a nursing home, and I’m looking at it from the vantage point of the government, what’s going to cost more? I might be able to give you a good deal of money for food as opposed to, I mean hey, we’re not going to solve this problem, I mean we’re giving away billions of dollars and money for food stamps to people who are using it to gamble with and everything else. We’re doing it, it’s like ‘*hey take some; you can have some*.’ I’m trying to project myself in the situation. If there were a way as a case worker that I could say, ‘*okay here’s something, here’s a situation I can apply some cash to make it possible for this person to live the way they want to for the few months they have left,*’ I think it would be a good thing...I think it deserves one”  Other woman: “Insurance companies are giving their workers the ability to choose what health they want. That’s all I’m saying, it’s the same; to me it’s the same thing. Cash let’s people choose how they get transportation or food”  Man 1: I’m kind of negative toward the government as well. But in this concept you have to give the benefit of the doubt that it will be administered in the way it was designed. If you don’ do that, you don’t get beyond where we are” |
| Session G | Man 1: “I think many people with a cancer diagnosis can cause them to lose their jobs and I think your ability to continue is dependent upon getting a little extra help”  Man 2: “I disagree with that. I think cash should not be part of an insurance program”  Man 3: “I agree with him as well with the fact of cash because cash to me - when you say cash, right away I think of the welfare program, the food stamps, the things of this nature…This is Medicare program, and when you say well, ‘*a person who has cancer is going to lose his job*,’ well if that person is working and he has the cancer, okay, usually companies can’t make you lose your job. All right, you still have insurance from the company, they can’t make you quit”  Man 2: “That’s not true because not all companies have insurance”  Man 3: “People that I know of that had cancer, and you know, they just take a leave of, not even a leave of absence. They’re there. Yes they won’t get their paycheck but once again, if you’re in a situation that you can’t work you get social security. I mean I’m looking at it – this is a Medicare program, and to me cash shouldn’t to the fact to say, ‘You have an illness, let me pay you for this”  “I don’t know what the original researchers intent was of putting cash in here, but they’re putting it in here as part of an exercise for us to say- and I’m interpreting it maybe differently than their intent, I’m not sure - I think I’m interpreting it in my mind as ‘*would people need some help with cash because you have cancer?*’ And this is a way of finding that. Whether it’s appropriate to come through Medicare, social security, or something else I’m not sure what the appropriate channel is, but I’m voting for yes people need cash.”  Man 2: “No. It opens up…chances of fraud are so big with cash”  Man 3: “Everybody needs cash, even if you have cash, you need cash. You’ll always want cash. I don’t think cash should come into play to make sure that the person is you know, taken care of. I’d rather see the money being going some place else.”  Moderator: There is no way to document where the cash is being spent”  (No agreement) |
