# Supplementary material for: A decision exercise to engage cancer patients and families in Deliberation about Medicare Coverage for advanced Cancer Care
Source: BMC Health Serv Res. 2014 Jul 19;14:315. doi: 10.1186/1472-6963-14-315 (PMC4112612; doi:10.1186/1472-6963-14-315)
Supplement: Additional file 5 — Verbatim comments from participants about how their own personal experience contributed to their reasoning in the deliberative process. Comments on personal experiences were presented in six of the 14 recorded sessions. Referenced in the text as Additional file 5. [file 1472-6963-14-315-S5.docx]

Additional File 5

| **Session** | **Verbatim Quotes Regarding Personal Experience** |
| --- | --- |
| Session H | “I go for the next treatment for cancer, the next level.” (Agreement)  “You have to know you’ve got a skewed population in here. Everybody is in treatment or has had treatment for cancer, or maybe you’ve seen it”  “I’ve experienced the consequences of it”  “That second level really takes you into some pretty serious philosophical issues” |
| Session B | “Well I’m going to throw out something before everything else is spent. Complementary. Because I use complementary alternative services – acupuncture and chiropractic- and so does my husband, for 25 years. It has basically kept us healthy, except for the fact that I got cancer” |
| Session G | “Speaking from my own personal experience, alright, because I had surgery. They cut all my muscles in my back, and therefore to try and get pain relief, okay, to get the blood flowing back there and whatever, they sent me to rehab. Well when you’re at rehab they pay for everything, you know at rehab. But rehab won’t give you a massage back there to try and stimulate that blood flow, so I have to go to a massage therapist; that’s all she works on is from here to my neck” |
| Session A | “I’m torn between the high level of cancer or the nursing facility. And then having personal experience with the nursing facility – not for me, but for family – I think I’d like to put two green circles on the basic level nursing. ‘*Pays for skilled nursing home or rehabilitation center, provides short term skilled nursing care or related services for patients. This can provide services to improve the function of injured, disabled, or sick persons. Such facilities provide all care needed by patients.’ And, ‘with basic two stickers, person receives help with daily needs, skilled care prescribed by the doctors, and provided by nurses and certified nurses’ assistants*.’” (Agreement on first level) |
| Session F | “We ought to put one in cosmetic care for those people who need wigs and those kind of things after chemo. Speaking from the breast cancer end of it, there are other ways to pay for it. (Moderators asks what other ways are) Well, there are, there’s like the Susan Komen, they’ll help you; there’s help out there. I didn’t have to pay a penny for my prosthesis arm”  “I kind of agree with her; I think there’s other sources for that”  I’m going to go with homecare. I just think that – so having worked with my mother when she was in her last years, the times that we, it was just so nice to be able to get away occasionally. And just talking about paying for somebody to come in and relieve the care giver“  “ Advice to me was always – that’s something somebody already should have done”  “Sometimes living wills and stuff, some people don’t face until they get to this position.  “We’ve been asked about ours 57 times in the last month. And I just said, ‘let me live,’ period”  “I think that’s important. Some people, it just depends on your – we talked about that; I think he said he’s in a support group. I didn’t see any need or want for it, but I think a lot of people do want that…I just didn’t want to go sit and listen to other people’s problems. I’m dealing with mine” |
